# Very Long-Chain Acyl-CoA Synthetase 3: Overexpression and Growth Dependence in Lung Cancer

**DOI:** 10.1371/journal.pone.0069392

**Published:** 2013-07-23

**Authors:** Zhengtong Pei, Peter Fraisl, Xiaohai Shi, Edward Gabrielson, Sonja Forss-Petter, Johannes Berger, Paul A. Watkins

**Affiliations:** 1 Hugo W. Moser Research Institute at Kennedy Krieger, Baltimore, Maryland, United States of America; 2 Department of Neurology, Johns Hopkins University School of Medicine, Baltimore, Maryland, United States of America; 3 Department of Pathology, Johns Hopkins University School of Medicine, Baltimore, Maryland, United States of America; 4 Center for Brain Research, Medical University Vienna, Vienna, Austria; H. Lee Moffitt Cancer Center & Research Institute, United States of America

## Abstract

Lung cancer is the leading cause of cancer deaths worldwide. In the United States, only one in six lung cancer patients survives five years after diagnosis. These statistics may improve if new therapeutic targets are identified. We previously reported that an enzyme of fatty acid metabolism, very long-chain acyl-CoA synthetase 3 (ACSVL3), is overexpressed in malignant glioma, and that depleting glioblastoma cells of ACSVL3 diminishes their malignant properties. To determine whether ACSVL3 expression was also increased in lung cancer, we studied tumor histologic sections and lung cancer cell lines. Immunohistochemical analysis of normal human lung showed moderate ACSVL3 expression only in bronchial epithelial cells. In contrast, all of 69 different lung tumors tested, including adeno-, squamous cell, large cell, and small cell carcinomas, had robustly elevated ACSVL3 levels. Western blot analysis of lung cancer cell lines derived from these tumor types also had significantly increased ACSVL3 protein compared to normal bronchial epithelial cells. Decreasing the growth rate of lung cancer cell lines did not change ACSVL3 expression. However, knocking down ACSVL3 expression by RNA interference reduced cell growth rates in culture by 65–76%, and the ability of tumor cells to form colonies in soft agar suspension by 65–80%. We also conducted studies to gain a better understanding of the biochemical properties of human ACSVL3. ACSVL3 mRNA was detected in many human tissues, but the expression pattern differed somewhat from that of the mouse. The enzyme activated long- and very long-chain saturated fatty acid substrates, as well as long-chain mono- and polyunsaturated fatty acids to their respective coenzyme A derivatives. Endogenous human ACSVL3 protein was found in a punctate subcellular compartment that partially colocalized with mitochondria as determined by immunofluorescence microscopy and subcellular fractionation. From these studies, we conclude that ACSVL3 is a promising new therapeutic target in lung cancer.

## Introduction

Acyl-CoA synthetases (ACS) catalyze the ATP-dependent thioesterification of fatty acids (FA) to coenzyme A (CoA) [1]. This “activation” step is necessary for FA to participate in nearly all subsequent metabolic reactions. Based on their acyl chain-length preference, as well as their amino acid sequence homology, the 26 different ACSs found in humans can be divided into several distinct families of enzymes, including the “very long-chain” (ACSVL) family, which contains 6 members. Five enzymes of the ACSVL family can activate long- to very long-chain FA substrates; the sixth member of this family is a liver-specific bile acid-CoA synthetase [2]. In addition to their metabolic functions, these enzymes have also been investigated as FA transport proteins (FATP) [3], as three of the six family members promote the cellular uptake of long-chain FA [4]. The official designation of the genes encoding the ACSVL/FATP family is *SLC27A1-6*.

We previously described properties of mouse ACSVL3 (Slc27a3; FATP3) [5]. When ACSVL3 was overexpressed in COS-7 cells, the protein localized to the endoplasmic reticulum. However, the endogenous enzyme in MA-10 mouse testis Leydig cells partially colocalized with mitochondria by both differential centrifugation and immunofluorescence. Transient knockdown of ACSVL3 using siRNA decreased the ability of MA-10 cells to activate either long-chain (palmitate; C16∶0) or very long-chain (lignocerate; C24∶0) FA. However, ACSVL3 knockdown did not affect the ability of MA-10 cells to take up C16∶0. The latter observation was subsequently confirmed when expression of mammalian ACSVL3 in yeast failed to promote long-chain FA uptake [4]. Northern blot and Western blot analyses of adult mouse tissues revealed that the enzyme is expressed primarily in adrenal gland, ovary, and testis, with weaker expression in other tissues such as brain, lung, and kidney [5]. High levels of ACSVL3 mRNA were present in embryonic (E12) mouse brain; however, expression rapidly decreased and by one month of age, the mRNA was barely detectable.

Adult mouse brain sections showed weak ACSVL3 immunostaining in cortical neurons, hippocampal neurons, and cerebellar Purkinje cells, but the protein was not detected in glial cells [5]. It was therefore unexpected when we found ACSVL3 to be markedly overexpressed in malignant gliomas and in human glioblastoma cell lines [6]. Knocking down ACSVL3 expression in human U87 glioblastoma cells decreased their malignant behavior both in culture and when injected either subcutaneously or intracranially in mice. Therefore, we asked whether ACSVL3 is expressed in other human malignancies such as lung cancer, which is the leading cause of cancer deaths worldwide [7]. In this paper, we show that ACSVL3 is highly overexpressed in all lung tumors and cell lines examined. In lung cancer cell lines, ACSVL3 depletion significantly improved their malignant growth properties. We also describe additional biochemical properties of human ACSVL3.

## Materials and Methods

### Materials

[1-^14^C]palmitic acid (C16∶0), [1-^14^C]oleic acid (C18∶1), [1-^14^C]arachidonic acid (C20∶4), [1-^14^C]docosahexaenoic acid (C22∶6) and [1-^14^C]lignoceric acid (C24∶0) were obtained from Moravek Biochemicals Inc. (Brea, CA, USA). [1-^14^C]linoleic acid (C18∶2) was from American Radiolabeled Chemicals (St. Louis, MO, USA). Polyclonal sheep antiserum to 70 kDa peroxisomal membrane protein (PMP70) was a gift from Dr. S. Gould (Johns Hopkins Univ. Sch. Med.). Mouse monoclonal antibody to protein disulfide isomerase and polyclonal rabbit antibody to manganese-superoxide dismutase (MnSOD), were from Stressgen (San Diego, CA, USA). Polyclonal rabbit antibody to ATP synthase was from Chemicon (CHEMICON International, Temecula, CA, USA). Polyclonal rabbit antibody to phosphatidylethanolamine N-methyltransferase 2 (PEMT), was a gift from Dr. D. Vance (University of Alberta, Edmonton, Canada). Affinity-purified polyclonal rabbit anti-ACSVL3 antibody was previously described [5]. HRP-conjugated secondary antibodies for Western blots were from either Bio-Rad Laboratories (Hercules, CA, USA) or Santa Cruz Biotechnology, Inc. (Santa Cruz, CA, USA), and secondary antibodies for immunofluorescence were from Jackson ImmunoResearch (West Grove, PA, USA). De-identified, formaldehyde-fixed paraffin sections of human lung cancer tissues from two patients were obtained and used in compliance with a protocol approved by the Johns Hopkins Office of Human Subjects Research Institutional Review Boards (Protocol NA_00003308); this protocol allows the use of de-identified tissues without additional written consent (other than that included in procedure consent forms). A tissue array (catalog # CBL-TMA-078) containing normal human lung and 67 different lung tumors was purchased from Creative BioLabs (Port Jefferson Station, NY).

### Cloning of Human ACSVL3 cDNA and Construction of Expression Plasmids

Full-length ACSVL3 sequence was obtained using a combinatorial approach of expressed sequence tag (EST) screening and the isolation of associated sequences using various cloning strategies. In brief, a single clone (C24355, Stratagene; GenBank Accession no. BC003041) that contained a long but 5′-unbounded open reading frame was used as a basis to obtain additional 5′ sequence, employing genomic primer walking. The subsequently identified 5′ extended genomic DNA sequence served to identify an additional EST clone, originating from a library enriched in full-length clones that had been obtained via the Cap-trapper method [8], located upstream of the first ATG in clone BC003041. This new EST clone (GenBank Accession no. BG720126), could be aligned with the genomic DNA and provided the novel cDNA sequence from position −115 to +179, which served as a template for further primer design in order to obtain full-length cDNA. A construct with a complete open reading frame of the human ACSVL3 gene was assembled in a two-step protocol. First, a 2480-bp *Hind*III-*EcoR*I fragment was excised from the cDNA clone C24355 and inserted into the expression vector pCDNA3.1(+) (Invitrogen), yielding p434. In the second step, a 915-bp PCR fragment was amplified from exon 1 of the genomic ACSVL3 clone, using forward primer, 549 (5′-CGCCAAGCTTAGCCAGATGCCTAAA-3′), with the ATG(+1) underlined, and reverse primer 530 (5′-AGCGCCACAGTTGCTCCAGGT-3′), purified, digested with *Hind*III (introduced with primer 549) and *Eco47*III, a unique restriction site in the ACSVL3 cDNA, and cloned into *Hind*III, *Eco47*III digested p434, resulting in plasmid p435, containing the full-length construct of ACSVL3. DNA sequencing was performed by MWG Biotech (Ebersberg, Germany).

### Cell Lines and Growth Conditions

Human HepG2 hepatoma, COS-1, and A549, H82, H460, EKVX, and U1752 lung cancer cell lines were from ATCC (Rockville, MD). HepG2 cells were maintained in minimal essential medium (Mediatech, Manassas, VA) supplemented with 10% fetal bovine serum (FBS; Gemini Bioproducts). COS-1 cells were grown in DMEM (Mediatech) supplemented with 10% FBS. All lung cancer cell lines were maintained in RPMI (Mediatech) supplemented with 10% FBS. Immortalized human bronchial epithelial cells (HBEC) [9] were generously provided by Dr. Jerry Shay (University of Texas Southwest Medical Center) and were maintained in serum-free BEGM (Bronchial Epithelial Growth Medium, Lonza, Walkersville, MD). All cells were cultured in a humidified incubator at 37°C under 5% CO_2_/95% air.

### Production of Clonal Lines with Stable ACSVL3 Knockdown and Measurement of Adherent and Anchorage-independent Cell Growth Rates

Clones with stable knockdown of ACSVL3 were produced using the pSilencer™ 4.1-CMV hygro vector (Ambion) as described previously [6]. Vectors containing knockdown sequences ACSVL3-3 (targeting nucleotides 397–415) and ACSVL3-4 (targeting nucleotides 1863–1881), which were both previously shown to decrease ACSVL3 expression in human cells, were transfected together into A549, EKVX, H82, and H460 lung cancer cell lines by electroporation. Control cells were transfected with a pSilencer vector (supplied by Ambion) that does not target any human mRNA sequence. Clones stably harboring control and knockdown plasmids were selected by hygromycin (250 µg/ml) resistance and analyzed for ASCVL3 expression by immunofluorescence and Western blot. Cell proliferation and anchorage-independent growth were measured as previously described [6].

### RNA Dot Blot and Northern Blot Analyses

A multiple tissue expression (MTE) array containing poly(A)-selected RNA from 61 different adult human tissues was obtained from Clontech. A cDNA probe was prepared by PCR amplification of a 657-bp fragment (position 1631 to 2287 of ACSVL3/SLC27A3 mRNA, NCBI accession # NM_024330) using 5′-GCACTGTATGGCCACATCTCC-3′ and 5′-TCTCTCAGGTGCCTCAGGTGT-3′ as forward and reverse primers, respectively, and plasmid p435 containing full-length ACSVL3 cDNA as template. To verify the specificity of the probe for ACSVL3, a BLAST search and multiple sequence alignments using the other members of the ACSVL/SLC27A family was performed and revealed no similarity to SLC27A1, 4, and 5, and only low similarity to SLC27A2 and 6 (not shown). For control, a 528-bp glyceraldehyde-3-phosphate dehydrogenase (GAPDH) probe was prepared by PCR using 5′-ACCACCATGGAGAAGGCTGG-3′ and 5′-CTCAGTGTAGCCCAGGATGC-3′ as forward and reverse primers, respectively. Conditions for probe labeling, hybridization, and detection were as described previously [10], [11]. Hybridization to the GAPDH probe was robust for all mRNA spots (not shown).

For Northern blot analysis, the same probe as for MTE array was hybridized to a human multiple tissue Northern blot (Ambion, Austin, TX, U.S.A.); a chicken β-actin probe was used as a control. Hybridization was carried out using Express Hybridization solution (Clontech) overnight at 65°C and washing at low stringency using 2×SSC, 0.05% SDS for 40 min at 65°C, followed by 0.1×SSC, 0.1% SDS for 40 min at 50°C for higher stringency. Hybridization of probes was detected by exposure to the Molecular Imager FX System (Bio-Rad).

### Subcellular Fractionation and Western Blot Analysis

HepG2 cells were fractionated as described previously [12]. Western blotting was performed as described previously [13]. For immunodetection mouse anti-MnSOD, rabbit anti-PEMT or rabbit anti-ACSVL3 primary antibodies were used along with HRP-conjugated anti-rabbit and anti-mouse secondary antibodies.

### Indirect Immunofluorescence and Immunohistochemistry

HepG2 cells grown to ∼60% confluence on glass coverslips were fixed in 4% formaldehyde in PBS and permeabilized with 1.0% Triton X-100 prior to incubation with primary (rabbit anti-ACSVL3, sheep anti-PMP70, mouse anti-protein disulfide isomerase, or rabbit anti-ATP synthetase) and secondary (FITC-conjugated anti-rabbit or Rhodamine-conjugated anti-mouse or anti-sheep IgG) antibodies as described previously [14]. Fluorescence was visualized using a Zeiss Axiovert epifluorescence microscope. Immunohistochemical detection of ACSVL3 in histologic sections of human lung tumors was as previously described [5], [6].

### Acyl-CoA Synthetase Assay

COS-1 cells were transfected with the ACSVL3 expression plasmid p435 or empty vector via electroporation as described previously [15]. Cells were harvested 3 days after transfection, washed, suspended in homogenization buffer, and were stored at −80°C prior to assay. Activation of [1-^14^C]-labeled FA (C16∶0, C24∶0, C18∶1, C18∶2, C20∶4 or C22∶6) to their CoA thioesters was performed as described previously [15]. Assays with C16∶0 contained 15 µg of COS cell protein, assays with C24∶0 contained 60 µg protein, and assays with all other FA contained 50 µg protein.

### Fatty Acid Composition

H460 and EKVX cells were grown to near confluence, harvested by gentle trypsinization, and washed with phosphate-buffered saline. Cell pellets containing 1.0 mg protein were extracted, derivatized with pentafluorobenzyl bromide, and quantitated by capillary gas chromatography-electron-capture negative-ion mass spectrometry (GC/MS) as described by Lagerstedt et al. [16]. Results are presented as percent of total fatty acids.

## Results

### Tissue Distribution of Human ACSVL3 mRNA

**Figure 1:**
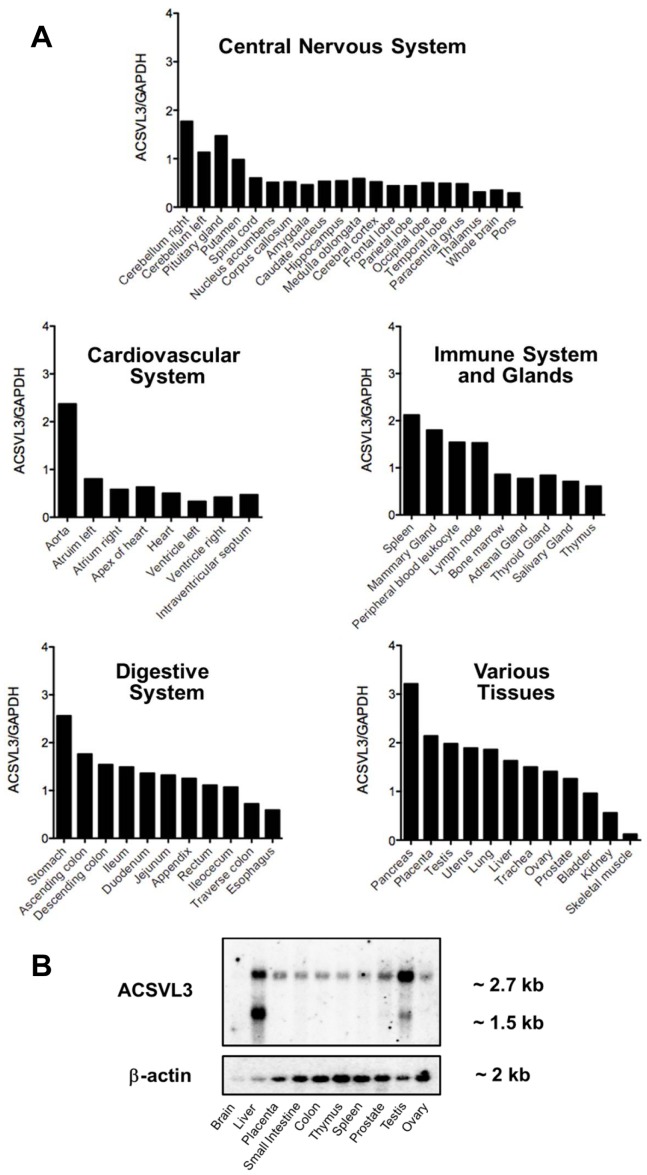
Expression profile of ACSVL3 mRNA in human tissues. (*A*) Human multiple tissue expression (MTE™) array RNA dot blot analysis. The RNA dot blot was hybridized with a ^32^P-labelled, 657-bp PCR fragment derived from the human ACSVL3 cDNA as described in Methods. The membrane was stripped and hybridized with a GAPDH probe to control for mRNA abundance. Signal intensity of individual dots was quantified using ImageJ software (available from: http://rsbweb.nih.gov/ij/download.html), and the ACSVL3 expression relative to that of GAPDH was calculated. (*B*) Human multiple tissue Northern blot containing 2 µg of poly(A)^+^-selected RNA was hybridized with the same ACSVL3 cDNA probe as in *(A)*. The lanes are: M, Millenium Markers™; 1, brain; 2, liver; 3, placenta; 4, small intestine; 5, colon; 6, thymus; 7 spleen; 8, prostate; 9, testis; and 10, ovary. Approximate sizes of the detected bands are shown to the right. Control hybridization with β-actin probe is shown in the bottom panel.

ACSVL3 mRNA expression was examined by RNA dot blot analysis using a multiple tissue expression (MTE™) array containing 61 adult human tissue samples (Fig. 1A). The signal intensity of each individual dot was quantified and ACSVL3 expression relative to that of GAPDH was calculated. The strongest normalized ACSVL3 signals were present in pancreas, stomach, aorta, and spleen. In the cardiovascular system, ACSVL3 mRNA was particularly enriched in the aorta, relative to all areas of the heart. The ACSVL3 transcript was relatively abundant throughout the digestive system, indicating a possible role in intestinal FA metabolism. The mRNA could also be easily detected in most lymphoid tissues, and in the reproductive system and urinary tract. Moderate signal intensity was obtained from most glands, except mammary gland and pituitary where the level appeared higher. Nearly all regions of the central nervous system, except for cerebellum and the pituitary gland, had very low ACSVL3 mRNA levels. ACSVL3 expression in skeletal muscle was also very weak. No signal indicative of non-specific binding of ACSVL3 probe to various control RNA and DNA samples was observed.

To establish the size of ACSVL3 mRNA, a human multiple tissue Northern blot was hybridized with the probe used for the MTE blot. This probe detected a 2.7 kb ACSVL3 transcript in all tissues except brain (Fig. 1B, top). The absence of a signal from the brain sample is likely due to the low amount of mRNA present in this lane, as documented by the β-actin control (Fig. 1B, bottom). The mRNA size is similar to that predicted from ACSVL3 cDNA. A second mRNA species of approximately 1.5 kb appeared in liver, testis and prostate (Fig. 1B, top). This smaller mRNA was most abundant in liver and therefore most likely contributed to the high signal in liver on the dot blot. The small size of this cross-hybridizing sequence would not be able to accommodate the open reading frame of a typical ACS and therefore was not analyzed further. Overall there is a good correlation of the expression levels between the Northern blot and multi tissue array.

### Acyl-CoA Synthetase Activity of Human ACSVL3

**Figure 2:**
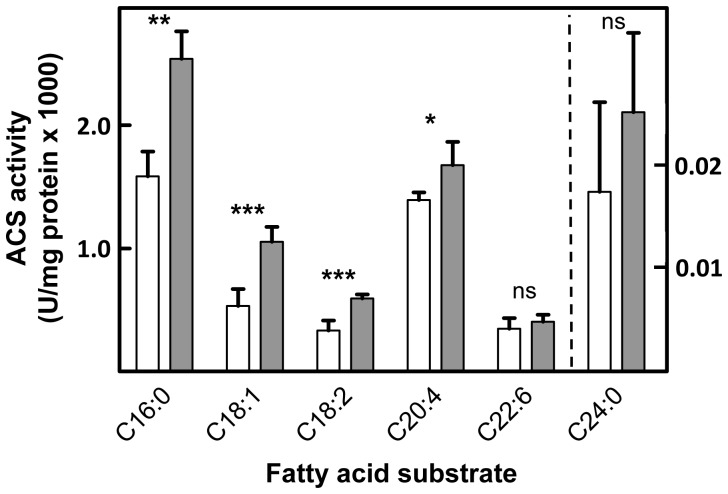
Acyl-CoA synthetase activity of human ACSVL3 protein. COS-1 cells overexpressing ACSVL3 (gray bars) from plasmid p435 (see Methods) and control cells transfected with empty vector (white bars) were assayed for ACS activity using the indicated ^14^C fatty acid as substrate as described in Methods. One unit (U) of enzyme activity = 1 µmol/min. Results are presented as the mean ± S.D. of three independent transfection experiments. ns, not significant. p-values: *<0.05; **<0.01; ***<0.001.

We analyzed the functional capability of human ACSVL3 protein to activate FA to their CoA thioesters following overexpression in COS-1 cells. Compared to COS-1 cells transfected with empty vector, cells expressing ACSVL3 showed increased ACS activity when assayed with several FA substrates. Statistically significant increases in activation of palmitic acid (C16∶0), oleic acid (C18∶1ω9), α-linoleic acid (C18∶2ω6), and arachidonic acid (C20∶4ω6) were observed (Fig. 2). Although we routinely measured increased capacity to activate the very long-chain FA, lignoceric acid (C24∶0), this did not reach statistical significance. A slight increase in activation of docosahexaenoic acid (C22∶6w3) was also observed, but was not statistically significant. Similar to what has been demonstrated for other ACSVL enzymes [15], [17], [18], overexpressed ACSVL3 protein activated long-chain FA to a greater extent than very long-chain FA.

### ACSVL3 Subcellular Localization in HepG2 Cells

**Figure 3:**
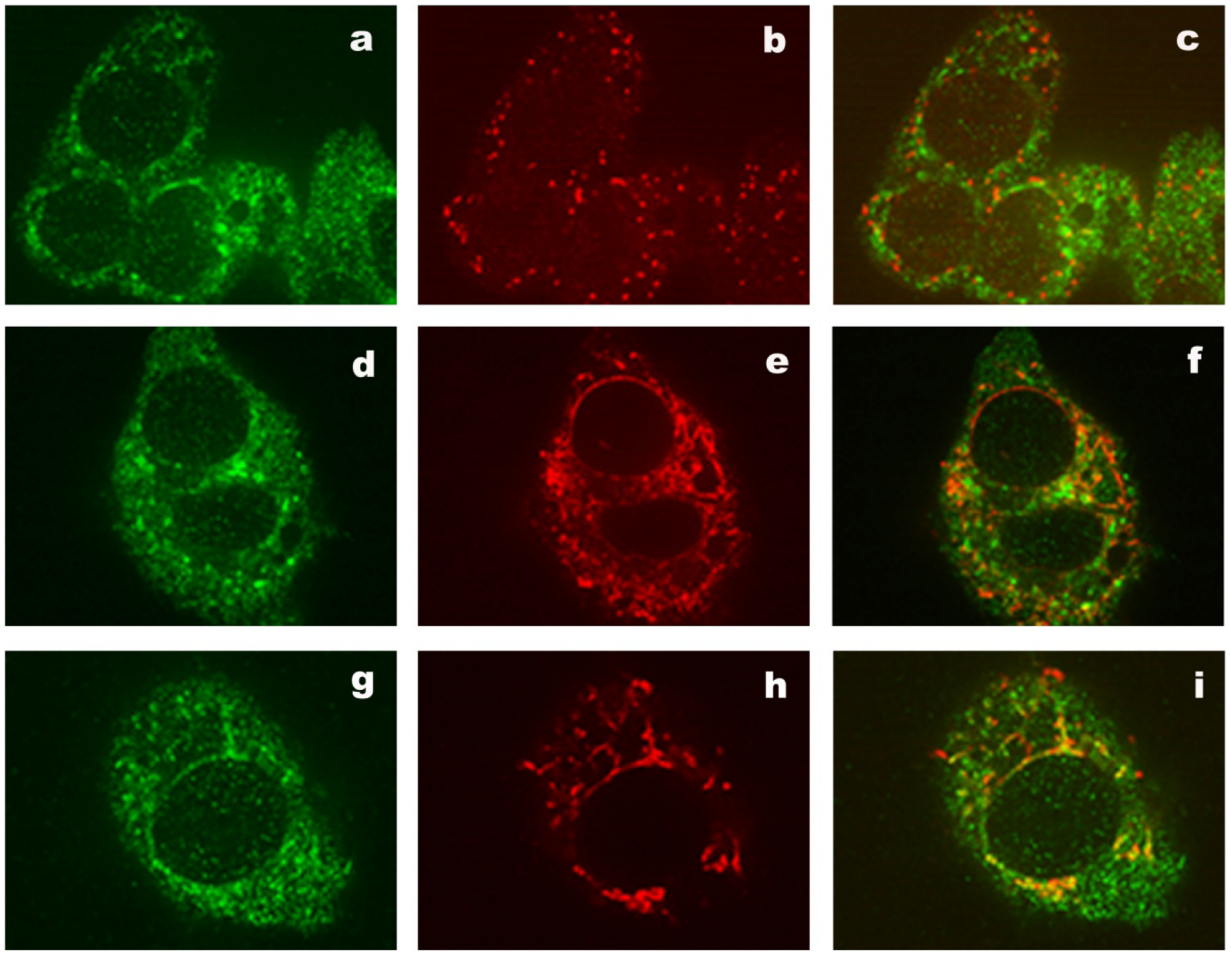
ACSVL3 immunofluorescence in HepG2 cells. Confocal microscopy was used to detect endogenous ACSVL3 in HepG2 cells by immunostaining with anti-ACSVL3 antibody (a, d, and g). Double-labeling with anti-ACSVL3 (a) and anti-PMP70 (b) demonstrated that the punctate ACSVL3-containing vesicles were not peroxisomes when the images were merged (c). Double-labeling with anti-ACSVL3 (d) and anti-protein disulfide isomerase (e) as a marker for endoplasmic reticulum revealed no colocalization in the merged image (f). Double-labeling using anti-ACSVL3 (g) and anti-ATP synthase (h) showed partial colocalization of ACSVL3 with mitochondria in the merged image (i).

Indirect immunofluorescence was used to localize endogenous ACSVL3 in the human hepatoma cell line HepG2. ACSVL3 exhibited a punctate fluorescence pattern when observed by confocal microscopy (Fig. 3a, d, and g). ACSVL3 immunostaining of these punctate structures did not colocalize with either the peroxisomal marker, PMP70 (Fig. 3b and c) or the endoplasmic reticulum marker, protein disulfide isomerase (Fig. 3e and f). ACSVL3 immunofluorescence colocalized partially with mitochondria visualized using ATP synthase as a marker (Fig. 3h and i).

**Figure 4:**
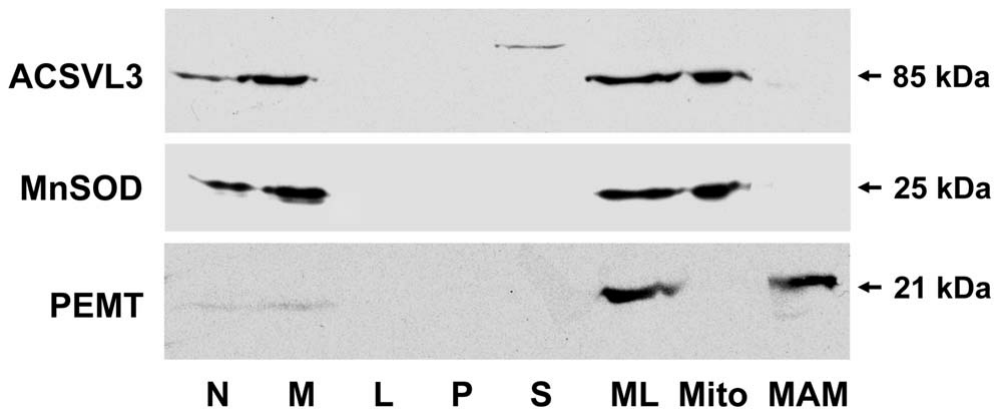
ACSVL3 localization in HepG2 cell subcellular fractions. HepG2 cells were fractionated into nuclear (N), mitochondrial (M), light mitochondrial (L), microsomal (P), and cytosolic (S) fractions by differential centrifugation, and a total mitochondrial (ML) fraction was further separated into purified mitochondrial (Mito) and mitochondria-associated membrane (MAM) fractions, as described in Methods. Each lane was loaded with ∼30 µg protein. Western blot analyses of the same membrane were performed using antibodies against ACSVL3, the mitochondrial marker Mn-superoxide dismutase (MnSOD), and the MAM marker phosphatidylethanolamine N-methyltransferase (PEMT).

To characterize further the subcellular localization of ACSVL3, HepG2 cells were fractionated by differential centrifugation. Homogenates were separated into fractions enriched in nuclei (N), mitochondria (M), peroxisomes (L), endoplasmic reticulum (P), and the membrane-free cytosol (S), and analyzed by Western blotting. ACSVL3 was found primarily in the M-fraction (Fig. 4), as indicated by the presence of the mitochondrial marker protein MnSOD. To a lesser extent the protein could also be detected in the N-fraction. No ACSVL3 signal could be detected in the L-, P-, or S-fractions. The presence of ACSVL3 in the N-fraction is likely due to contamination with incompletely homogenized cells, as MnSOD and phosphatidylethanolamine N-methyltransferase 2 (PEMT) were also detected in this fraction (Fig. 4).

Because colocalization of ACSVL3 and mitochondria by immunofluorescence was not complete, we speculated that ACSVL3 might be found in the mitochondria-associated membrane (MAM) fraction, an endoplasmic reticulum-derived compartment that sediments with mitochondria during differential centrifugation [19]. Therefore, we resolved the MAM fraction from purified mitochondria by centrifugation through Percoll. The MAM-specific marker protein, PEMT, was detected in the MAM fraction only, demonstrating the successful separation of mitochondria and MAM. There was a significant enrichment of the ACSVL3 signal in purified mitochondria, but the protein was not detectable in the MAM fraction (Fig. 4). As we observed only partial colocalization of ACSVL3 with mitochondria by immunofluorescence analysis (Fig. 3), despite its sedimentation in this fraction and its absence from the MAM fraction, we propose that the ACSVL3 protein resides in a novel subcellular compartment that is distinct from but tightly associated with mitochondria. A similar result was obtained for murine ACSVL3 [5].

### ACSVL3 Expression in Normal Human Lung and in Lung Tumors

**Figure 5:**
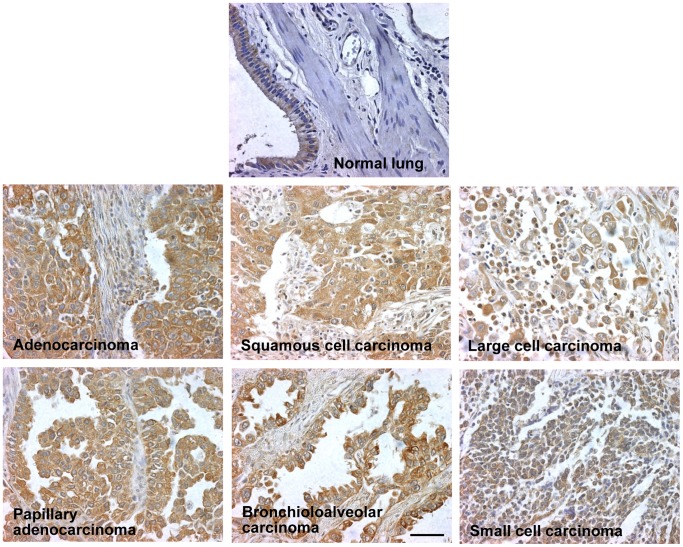
ACSVL3 expression in normal human lung and lung tumors by immunohistochemistry. Paraffin sections of lung tumors present on a tissue array were immunostained with anti-ACSVL3 antibody (brown) and counterstained with hematoxylin (blue). Tumors representative of the 67 different tumors present on the array, as well as normal lung tissue, are shown. All tumors stained positive for ACSVL3. Total magnification, 400×. Bar = 50 µm.

We previously reported that, despite weak expression in brain and essentially no detectable protein in glia, ACSVL3 levels were robustly elevated in malignant glioma and in human glioblastoma cell lines [6]. To determine whether other malignancies overexpressed ACSVL3, we examined expression of this protein in normal human lung and in lung tumors by immunohistochemistry. Modest ACSVL3 expression was observed in normal bronchial and bronchiolar epithelial cells (Fig. 5). No ACSVL3 histochemical staining was seen in type I or type II alveolar cells, goblet cells, stromal cells, or cells comprising the pulmonary vasculature. In contrast, tissue sections from tumors resected from two patients at the Johns Hopkins Hospital, one diagnosed as adenocarcinoma and the other as squamous cell carcinoma, exhibited highly elevated ACSVL3 levels (not shown). To confirm these initial observations, we performed immunohistochemical analysis on a tissue array containing 67 different human lung tumors. Represented on the array were 25 squamous cell carcinomas, 21 adenocarcinomas, 6 papillary adenocarcinomas, 7 small cell carcinomas, 3 large cell carcinomas, one bronchioloalveolar carcinoma, one basaloid squamous cell carcinoma, and 3 carcinoid tumors. Of these tumors, 100% showed overexpression of ACSVL3; several representative tumors are shown in Fig. 5. There was no obvious correlation between extent of ACSVL3 expression and the differentiation state of the tumor.

### ACSVL3 Expression in Lung Tumor Cell Lines

**Figure 6:**
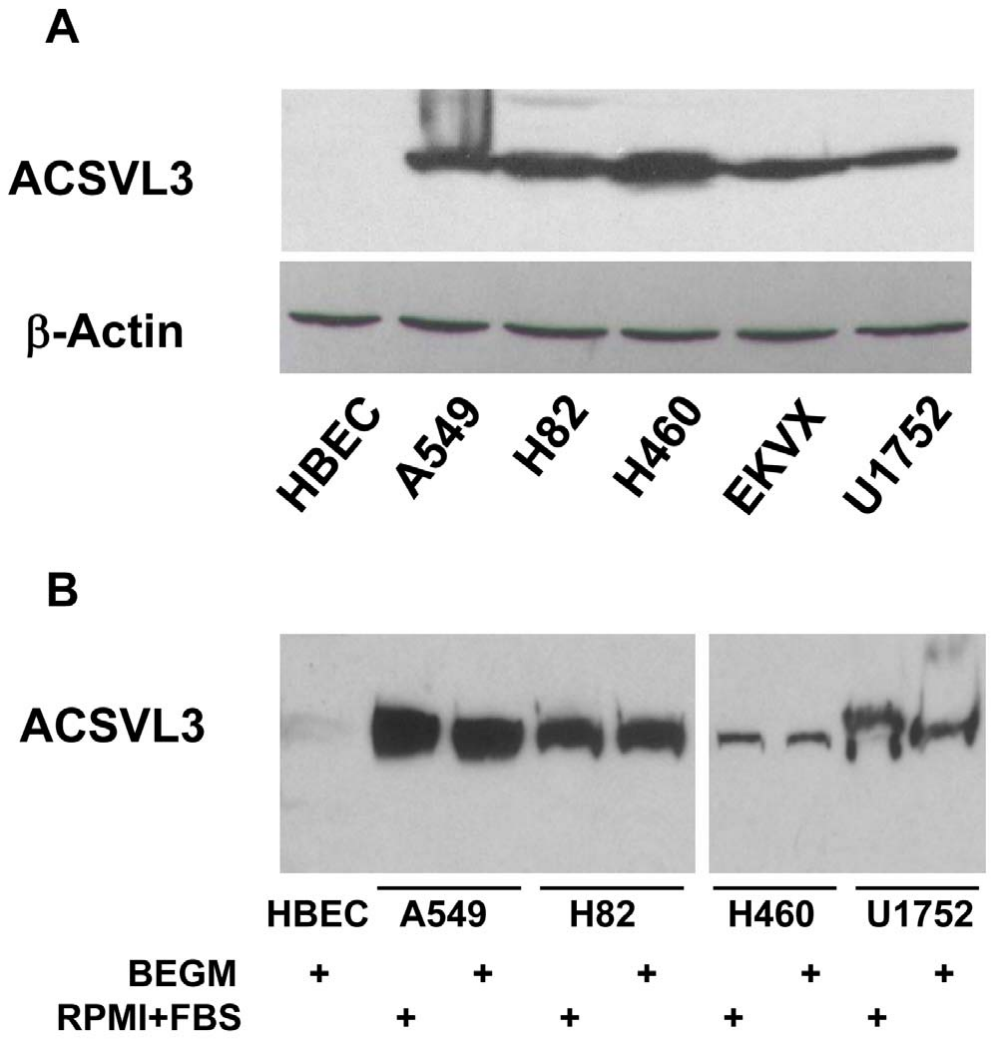
ACSVL3 expression in human bronchial epithelial cells and lung cancer cell lines. (*A*) Growth under standard culture conditions. HBEC and five lung cancer cell lines were cultured as described in Methods and analyzed for ACSVL3 expression by Western blot. Tumor cell lines were derived from non-small cell carcinoma (A549), small cell carcinoma (H82), large cell carcinoma (H460), adenocarcinoma (EKVX), and squamous cell carcinoma (U1752). β-Actin expression was determined as a loading control. (*B*) Effect of growth rate reduction on ACSVL3 expression in lung cancer cell lines. HBEC, cultured in serum-free BEGM, grow significantly slower than cancer cell lines. To slow their growth rates, A549, H82, H460, and U1752 cells were transferred to BEGM. All cells were maintained in this medium for 3 weeks, at which time they were harvested and subjected to Western blot analysis. Tumor cells grown in standard medium, RPMI plus 10% fetal bovine serum (FBS), were harvested for comparison. Growth rates in BEGM for all cancer cell lines were decreased by more than 50%.

To confirm and extend these observations, we examined ACSVL3 expression in normal human bronchial epithelial cells (HBEC), and in several lung cancer cell lines. ACSVL3 expression was barely detectable in HBEC (Fig. 6A and 6B). Cell lines derived from human lung tumors, representing small cell carcinoma (H82), non-small cell carcinoma (A549), adenocarcinoma (EKVX), squamous cell carcinoma (U1752), and large cell carcinoma (H460), all robustly expressed ACSVL3 (Fig. 6A).

HBEC grow at a slow rate as they are cultured in serum-free synthetic medium (BEGM) to prevent squamous differentiation [20]. Because the tumor cell lines, cultured in rich medium containing fetal bovine serum, grow significantly faster than HBEC, we asked whether high ACSVL3 levels were simply a consequence of the rapid growth rate. Four tumor cell lines, A549, H82, H460, and U1752, were transferred to serum-free BGEM and were maintained in this medium for three weeks. Despite a >50% decrease in growth rate, these cell lines all maintained high ACSVL3 expression (Fig. 6B). This observation suggests that factors other than growth rate are responsible for maintenance of high ACSVL3 levels in cancer cell lines.

### Effect of ACSVL3 Knockdown on Adherent and Non-adherent Growth of Lung Tumor Cell Lines in Culture

**Figure 7:**
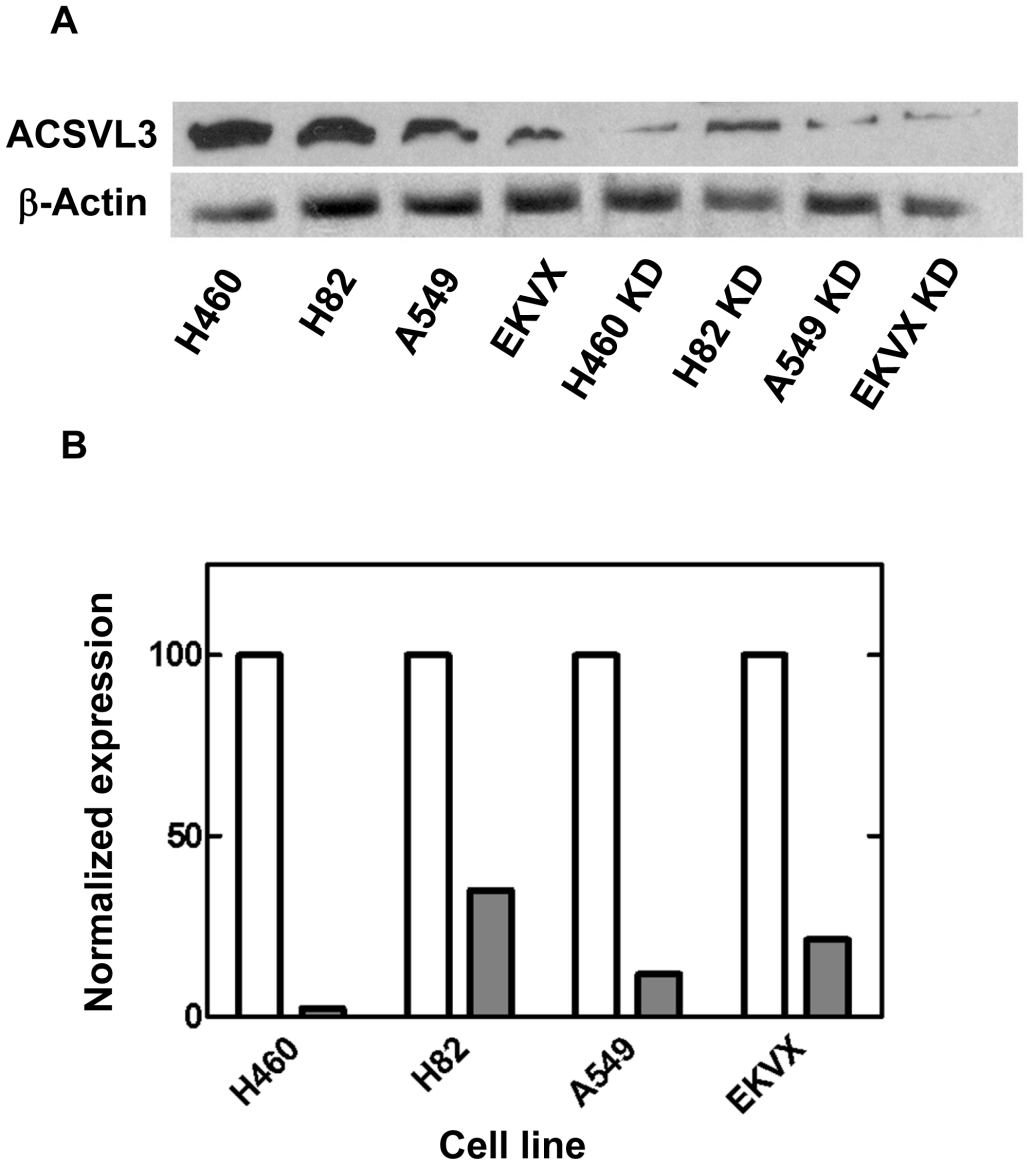
ACSVL3 knockdown in lung cancer cell lines. The short hairpin-producing vector pSilencer (Ambion) was used to generate several lung cancer cell lines with decreased ACSVL3 expression as described in methods. Clonal lines of H460, H82, A549, and EKVX lung cancer cells with stable knockdown (KD) of ACSVL3 were selected. Control lines stably harbor a plasmid containing a scrambled sequence that does not target any human or rodent mRNA. (*A*) ACSVL3 expression in control and KD cells was assessed by Western blotting. (*B*) Gel-Pro Analyzer 4.0 software was used to quantitate the Western blot signals in (A). Expression of ACSVL3 relative to β-actin was calculated for each pair of control and KD cell lines. Control cell line ratios were arbitrarily set to 100 and the relative, normalized expression of ACSVL3 in KD cells was calculated. White bars, control cells; grey bars, KD cells.

Stable knockdown of ACSVL3 expression in glioblastoma cells decreased their malignant phenotype in culture [6]. Both growth on culture dishes and the ability to form colonies in soft agar suspension were more characteristic of normal cells when ACSVL3 was depleted. To determine whether ACSVL3 expression also influenced growth of lung cancer cells, we produced four lines with stable ACSVL3 knockdown using RNA interference as described in Methods. Control cell lines stably harboring a plasmid encoding a scrambled short hairpin RNA were also produced. All four knockdown cell lines had decreased ACSVL3 protein levels by Western blot (Fig. 7).

**Figure 8:**
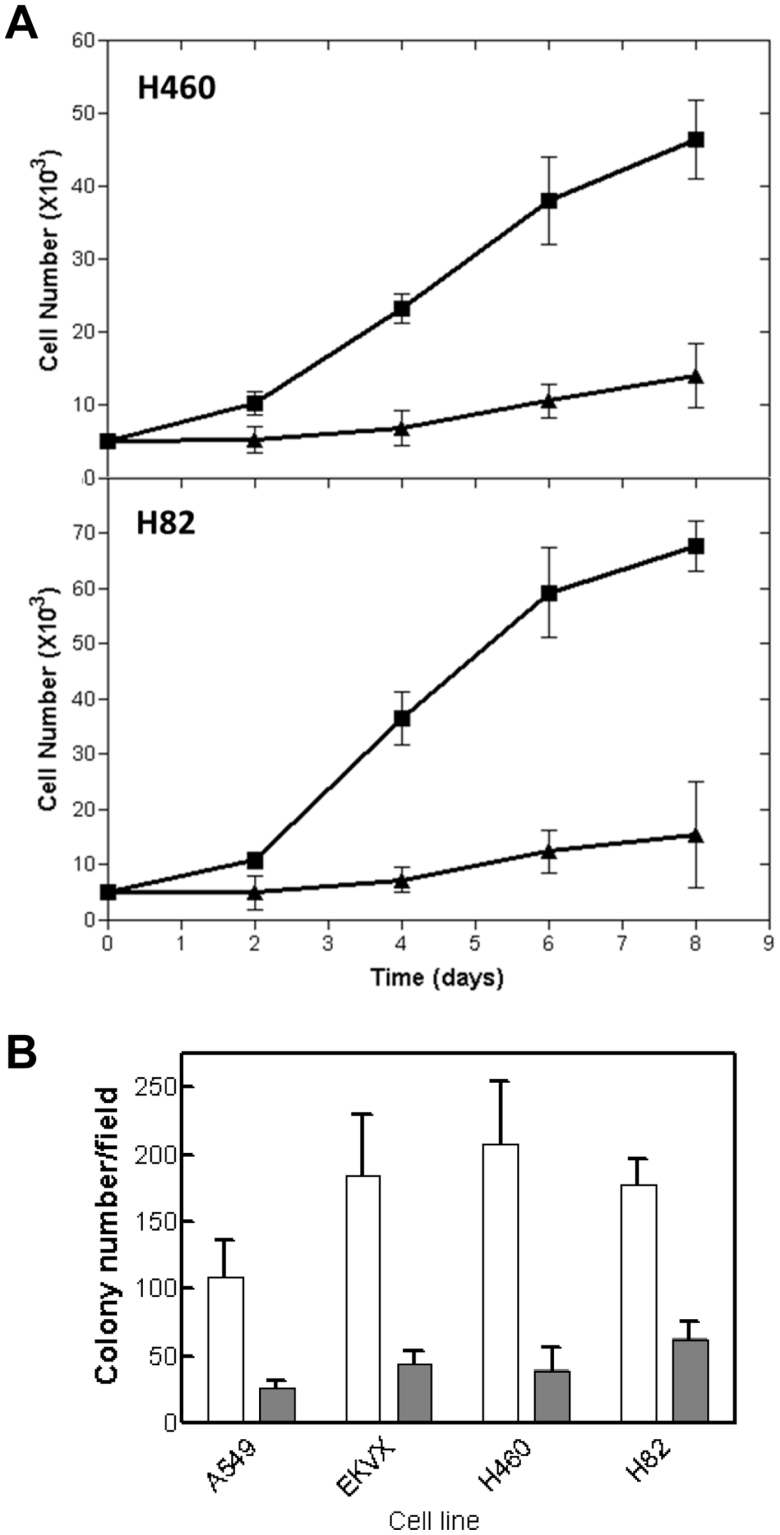
Effect of ACSVL3 knockdown on adherent and non-adherent growth rates of lung cancer cell lines. (*A*) Growth in culture. Control (▪) and knockdown (▴) H460 and H82 cells (5×10^3^) were seeded into 6-well plates. On days 2, 4, 6, and 8, cells from triplicate wells were harvested by trypsinization and counted using a hemacytometer. Mean ± S.D. is plotted. (*B*) Anchorage-independent growth. Control (white bars) and KD (gray bars) A549, EKVX, H460, and H82 cells (5×10^3^) were mixed with soft agar and seeded into 6-well plates. On day 20, cells in triplicate wells were stained with trypan blue and the number of colonies per field assessed by light microscopy. Mean ± S.D. is plotted. Differences between control and knockdown for all four cell lines were significant (p<0.01).

We then measured the effect of ACSVL3 knockdown on proliferation of cultured H460 and H82 lung cancer cell lines. As shown in Fig. 8A, lack of ACSVL3 decreased cell growth rates by 65–76% (measured on day 6). We also measured non-adherent growth of four cell lines, A549, EKVX, H82, and H460, in soft agar suspension. As shown in Fig. 8B, colony formation was decreased in all ACVL3-deficient cell lines by 65–80%. These findings indicate that ACSVL3 depletion in lung cancer cells, as in glioblastoma cells, decreases their malignant *in vitro* growth phenotype.

### Effect of ACSVL3 Knockdown on Cellular FA Composition

To begin to understand the role of ACSVL3 on tumor cell lipid metabolism, we analyzed the FA composition of control and ACSVL3 knockdown H460 and EKVX cells. No substantive differences in long-chain saturated FA containing 14–18 carbons between control and ACSVL3 knockdown were observed for either cell line (Table 1). Saturated very long-chain FA levels (24–26 carbons) were somewhat lower in both H460 and EKVX knockdown cells relative to controls. Interestingly, monounsaturated FA of both the n-9 and n-7 series were generally higher in knockdown H460 cells, but not in EKVX cells, compared to their respective controls; however, palmitoleic acid (C16∶1(n-7)) was reduced in EKVX cells with ACSVL3 knockdown. Polyunsaturated FA of both the n-6 and n-3 series were lower in H460 cells following ACSVL3 knockdown. Arachidonic acid (C20∶4(n-6)) levels were increased by ACSVL3 knockdown in EKVX cells, but levels of other polyunsaturated FA were relatively unchanged.

**Table 1:** Fatty acid composition of control and ACSVL3-depleted lung cancer cell lines.

Fatty Acid	H460 Control	H460 Knockdown	EKVX Control	EKVX Knockdown
**Saturated**				
C14∶0	1.9	2.2±0.1	0.94	0.83
C16∶0	18.8	18.4±0.2	20.6	19.0
C18∶0	17.8	16.6±1.1	14.3	15.6
C24∶0	1.6	1.3±0.1	5.1	4.6
C26∶0	0.12	0.09±0.01	0.23	0.16
**Monounsaturated**				
C18∶1(n-9)	20.2	27.5±2.1	16.6	15.0
C22∶1(n-9)	0.09	0.15±0.01	0.04	0.05
C24∶1(n-9)	0.8	1.1±0.0	1.9	1.8
C16∶1(n-7)	2.0	3.9±1.2	3.2	1.2
C18∶1(n-7)	4.4	5.9±0.3	4.9	3.9
**Polyunsaturated**				
C18∶2(n-6)	3.3	2.0±0.4	3.6	3.8
C20∶4(n-6)	11.1	6.8±1.3	11.0	15.3
C18∶3(n-3)	0.06	0.03±0.01	0.04	0.04
C20∶5(n-3)	1.3	0.7±0.1	2.4	2.5
C22∶6(n-3)	3.4	2.9±0.1	4.5	4.2

Cells were grown to near confluence prior to harvest. Lipids were extracted, hydrolyzed, and FA converted to their pentafluoobenzyl bromide derivatives prior to quantitation by GC-MS as described in Methods. For H460, three independent knockdown clones were averaged; for EKVX, two independent knockdown clones were averaged. Results are presented as percent of total FA (± SEM for H460 knockdown).

## Discussion

Although ACSs catalyze a fundamental reaction in cellular metabolism, the specific physiological function(s) of each of the 26 human ACS enzymes are only beginning to be elucidated. Based on tissue and cell expression pattern, subcellular location, substrate specificity, and other properties, we and others have suggested that each ACS likely plays a relatively specific role by channeling the acyl-CoA reaction product toward a particular metabolic fate [1], [21].

While physiological effects of gene manipulation in mice cannot always be extrapolated to humans, studies of knockout (KO) and transgenic mice have begun to address the functions of several members of the very long-chain ACS subfamily that includes ACSVL3. KO of ACSVL1 (Slc27A2; FATP2) was associated with decreased peroxisomal β-oxidation of very long-chain FA in liver and kidney [22]. Mice with cardiac-specific overexpression of FATP1 (Slc27a1; ACSVL4) at around 3 months of age developed lipotoxic cardiomyopathy and other pathologic features seen in diabetes [23]. FATP1 KO mice were phenotypically normal when fed a regular chow diet, but were protected from insulin resistance and accumulation of fatty acyl-CoA in muscle when placed on a high-fat diet [24]. Further studies showed that FATP1 KO mice had little or no insulin-stimulated FA uptake in skeletal muscle and adipose tissue [25]. These studies suggest a potentially important role for FATP1 in diabetes, obesity, and the metabolic syndrome. FATP4 (Slc27a4; ACSVL5) KO mice were characterized primarily by skin abnormalities, including decreased barrier function and a restrictive dermopathy that was lethal on postnatal day 1 [26], [27]. Mutations in FATP4 cause Ichthyosis prematurity syndrome in humans [28]. One member of the very long-chain ACS subfamily thioesterifies CoA to bile acids rather than to FA substrates [29], [30]. KO of this bile acid-CoA synthetase (BACS; FATP5; Slc27a5) in mice produced defects in bile acid conjugation [31]. Consistent with this observation was the identification of a patient with a homozygous mutation in a catalytically critical region of ACSB [32]. Interestingly, mice with ACSB KO also had alterations in hepatic FA metabolism. Hepatic triglycerides and free FA were decreased despite increased expression of fatty acid synthetase; furthermore, there was a redistribution of lipids from liver to other long-chain FA-metabolizing tissues [33].

To date, ACSVL3 is the only member of the very long-chain ACS family that has been associated with cancer in humans [6]. Elevated levels of ACSL4, a member of the long-chain ACS family, has been reported in carcinomas of the colon [34] and liver [35], and in hormone receptor-negative cancers of the prostate and breast [36]. Another long-chain ACS, ACSL5, was elevated in high-grade gliomas [37]. In contrast, lower levels of ACSL5 were reported in bladder cancer [38]. The mechanistic associations between ACS expression and malignancy have not been fully characterized.

Both overexpression and knockdown studies have shown that ACSVL3 catalyzes the formation of fatty acyl-CoAs [5], [6]. Although the related proteins ACSVL1, FATP1, and FATP4 have been shown to promote the cellular uptake of long-chain FA in addition to their ACS activity [4], several studies failed to show ACSVL3-dependent FA uptake [4], [5]. In contrast, Hagberg et al. recently reported that vascular endothelial growth factor-B (VEGFB) increased ACSVL3 and FATP4 expression and promoted FA uptake in primary endothelial cells [39]; mice null for VEGFB had decreased lipid accumulation in muscle, heart, and brown adipose tissue, and increased accumulation in white adipose tissue.

In this report, we show that several biological properties of human ACSVL3 are similar to those previously reported for the mouse enzyme [5]. Both enzymes activate long- to very long-chain FA substrates. Both human and mouse endogenous ACSVL3 proteins are found in a punctate subcellular compartment that partially colocalizes with mitochondria as determined by subcellular fractionation as well as microscopically. ACSVL3 mRNA was present in many tissues, but some differences between the two species in expression pattern were noted. For example, expression in mouse liver and lung was weaker than in the corresponding human tissues. Expression in adult brain was generally weak in both species.

Results reported here demonstrate that ACSVL3 is highly over-produced in all types of human lung tumors, including adeno-, squamous cell, non-small cell, large cell, and small cell carcinomas. All of 69 tumors examined (67 on the tumor array, and two from Johns Hopkins Pathology) exhibited rather robust immunostaining. This observation is similar to that made previously with a human glioma tissue array [6], and suggests that ACSVL3 overexpression may be a feature of other human cancers as well. Fatty acid synthase (FASN) and carnitine palmitoyltransferase 1c (CPT1C) are FA metabolism enzymes that have also been reported to be elevated in lung cancer [40], [41], [42]. Although both are overexpressed in a high percentage of lung tumors, neither FASN nor CPT1C levels were elevated in all tumors examined.

Cell lines representing the major types of lung tumors present on the tissue microarray were investigated, and ACSVL3 was found to be highly overexpressed in all cell lines tested. All cell lines are well-characterized and exhibit in vitro malignant features such as rapid growth and the ability to form colonies in soft-agar suspension. Slowing the growth rate of these cancer cells by maintaining them in a serum-free medium was not sufficient to decrease ACSVL3 levels. In contrast, depleting lung cancer cells of ACSVL3 significantly decreased their rapid growth rates, and impaired their ability to grow in suspension culture. These observations, along with similar findings in human glioblastoma cell lines [6], support our contention that ACSVL3 may be a unique therapeutic target in cancer.

The role of ACSVL3 in lipid metabolism has not been fully elucidated. To begin to address this, we examined the fatty acid composition of control and ACSVL3 knockdown H460 and EKVX cells. We found decreased levels of C26∶0, consistent with the proposed role for ACSVL family enzymes in saturated very long-chain FA metabolism. Interestingly, ACSVL3 knockdown affected the cellular levels of n-7 monounsaturated FA. Surprisingly little is known about the metabolic functions of n-7 FA in either normal or cancer cells; our results suggest that further investigation is warranted. Decreased ACSVL3 expression had somewhat different effects on levels of other FA in the two cell lines, indicating that this enzyme may play cell-specific roles in cancer cell lipid metabolism.

Lung cancer remains the leading cause of cancer deaths in the U.S. [7]. The costs of this disease in terms of human suffering, strain on healthcare system resources, and overall economic burden are staggering. Despite advances in treatment, overall five year survival rates have improved only slightly over the past 30 years, and remain at 16% [7]. Thus, new paradigms that lead to new treatment strategies are urgently needed. Pharmacological targeting of ACSVL3 may represent one such strategy. The mechanism(s) by which high levels of ACSVL3 support malignancy is unknown. We hypothesize that ACSVL3 supports malignancy by altering tumor cell metabolism. Studies aimed at identifying these metabolic changes are currently underway and may reveal additional drug targets in lung and other cancers.
